# Intramedullary Nailing for Humeral Shaft Fractures: Functional Outcome Assessment Within a Cohort of 202 Patients

**DOI:** 10.3390/jcm14217782

**Published:** 2025-11-02

**Authors:** Alessandro Zanzi, Pietro Maniscalco, Edoardo Fantinato, Gianfilippo Caggiari, Giorgio Moretti, Michele Francesco Surace, Corrado Ciatti

**Affiliations:** 1 Orthopedics and Traumatology Department, Viale San Pietro 24, 07100 Sassari, Italy; a.zanzi@studenti.uniss.it (A.Z.); gcaggiari@uniss.it (G.C.); 2 Department of Medicine and Aging Sciences, University of Chieti-Pescara, 66100 Chieti, Italy; pietro.maniscalco@unipr.it; 3 IRCCS Ospedale Galeazzi—Sant’Ambrogio, 20157 Milan, Italy; giomore95@yahoo.it; 4 Department of Orthopaedic and Trauma Sciences M. Boni, Universitas Studiorum Insubriae, 21100 Varese, Italy; 5 Orthopedics and Traumatology Department, Guglielmo da Saliceto Hospital, 29121 Piacenza, Italy; 6 Department of Medicine and Surgery, University of Parma, 43126 Parma, Italy

**Keywords:** antegrade intramedullary nailing, constant score, DASH, functional outcome, humeral shaft fracture, radial nerve palsy

## Abstract

**Background**: Humeral shaft fractures (HSFs) represent 13–25% of humeral fractures and are frequently complicated by radial nerve palsy and nonunion. While conservative management was historically preferred, surgical fixation with intramedullary nailing (IMN) has gained increasing popularity. The aim of this study was to evaluate the effectiveness of IMN in the treatment of HSFs, focusing on postoperative complications and functional outcomes. **Methods**: A bicenter retrospective analysis was conducted on 202 patients who underwent antegrade IMN fixation for HSF between 2014 and 2019, with a minimum follow-up of four years. Demographic data, trauma characteristics, surgical details, and postoperative complications were recorded. Functional outcomes were assessed at one year using the Disabilities of the Arm, Shoulder and Hand (DASH), Oxford Shoulder Score (OSS), Constant Shoulder Score (CSS), and Visual Analogue Scale (VAS). Statistical analysis included ANOVA, Student’s *t*-test, Spearman’s correlation, and multivariate regression. **Results**: The mean follow-up was 57.7 ± 19.6 months. At one year, mean OSS, DASH, CSS, and VAS scores were 39.0, 16.6, 73.5, and 0.9, respectively. Excellent or good Constant outcomes were recorded in 89.6% of patients. Sixteen complications (7.9%) occurred, including nerve injuries (4.0%) and pseudoarthrosis (1.5%). Patients operated within 48 h had significantly better functional scores compared to those treated later (*p* < 0.01). No differences were found according to fracture pattern, sex, diabetes, or osteoporosis. Age showed a weak correlation with functional outcomes. **Conclusions**: IMN is a safe and effective option for the treatment of HSFs, with high rates of functional recovery and a low incidence of complications. Early surgical intervention appears to improve outcomes, supporting its role as a valuable strategy in HSF management.

## 1. Introduction

The humeral shaft is defined as the portion of the humerus located between the insertion of the pectoralis major and the supracondylar ridge [1]. Humeral shaft fractures (HSFs) are relatively uncommon, with an incidence ranging between 1 and 3% of all fractures; among humeral fractures, they account for approximately 13–25% [2,3,4]. Recent studies have demonstrated a bimodal age distribution, with a first peak between 20 and 30 years of age and a second, larger peak between 70 and 80 years [5]. The mechanisms of injury vary with age: high-energy trauma is more frequent in younger patients, whereas low-energy trauma is more typical in the elderly [6]. Female sex and osteoporosis are considered the main risk factors [5,6].

At present, the most widely adopted classification for HSF is the Orthopaedic Trauma Association (OTA)/Arbeitsgemeinschaft für Osteosynthesefragen (AO) system, which provides information regarding the fracture pattern and the presence of comminution [7]. Owing to its anatomical location, this fracture type is frequently complicated by radial nerve palsy (RNP), particularly when the lesion involves the distal third of the shaft [2,5,8,9,10]. In a systematic review of 4517 cases, Shao et al. reported an incidence of RNP of 11.8% [11]. High-energy trauma and open fractures are recognized risk factors for primary RNP, while secondary or iatrogenic RNP may develop after surgical treatment, representing 10–20% of all cases associated with HSF [12].

Historically, conservative management with functional bracing was the preferred treatment [1]. However, the increasing incidence of HSF and the importance of related complications, combined with advances in surgical techniques and lower patient tolerance for residual deformities, have shifted the therapeutic approach toward surgical fixation [6]. Among surgical options, intramedullary nailing (IMN), both antegrade and retrograde, and plating are the most widely used techniques, although there is still no consensus on which method should be considered superior [6,13,14,15,16,17]. Only a limited number of studies have specifically analyzed secondary radial nerve palsy (RNP) occurring after intramedullary nailing of humeral shaft fractures, and the reported incidence varies between 2% and 6% [8,10,18]. During IMN fixation, RNP may arise as a consequence of fracture manipulation, intramedullary reaming, or distal locking [8,10,18].

Late complications include infection and nonunion. Infection is a potential adverse event in any surgical procedure, with an incidence of 1.6% in this context [19]. Identification of the responsible pathogen is essential, as it allows for targeted antibiotic therapy and, when necessary, the removal of the implant and infected tissue followed by external fixation. Pseudoarthrosis (PSA) is defined as the absence of fracture healing approximately six months after trauma. It may be hypertrophic or atrophic, depending on the presence or absence of callus formation [20]. The incidence ranges between 8 and 12%, which makes the humeral shaft one of the sites most at risk. Most cases of PSA occur in transverse fractures of the middle third of the shaft.

The aim of the present study was to evaluate whether IMN can be considered an effective surgical option for HSF. The primary endpoint was the incidence of postoperative complications, while the secondary endpoint was the functional outcome, assessed through both objective and subjective scales.

## 2. Materials and Methods

This bicenter retrospective study was conducted at Guglielmo da Saliceto Hospital (Piacenza, Italy) and Circolo ASST Sette Laghi Hospital (Varese, Italy). All patients who underwent surgical fixation of HSF with long IMN between 1 January 2014 and 31 December 2019 were considered eligible. Follow-up was extended until 31 December 2023. Patients were eligible for inclusion if they presented with an acute, isolated humeral shaft fracture of non-pathologic origin, confirmed radiographically, and were treated with antegrade intramedullary nailing. Both high- and low-energy trauma mechanisms were accepted. Eligible patients were aged between 20 and 79 years and provided a minimum follow-up of 48 months. Exclusion criteria included open fractures Gustilo–Anderson grade II or higher, pathological fractures, polytrauma cases requiring damage-control fixation, pre-existing neurological deficits involving the injured limb, delayed surgery beyond 15 days. Patients with incomplete clinical data or insufficient follow-up were also excluded. Clinical records were reviewed to collect information on fracture side, comorbidities, time from admission to surgery, operative time, length of hospital stay, and early complications.

A total of 238 patients with humeral shaft fractures treated with intramedullary nailing were initially identified from institutional databases of the two centers. After applying the inclusion and exclusion criteria, 202 patients were included in the final analysis. Exclusions consisted of 14 patients with incomplete follow-up data, 7 with open fractures, 6 with pathological fractures, and 3 with concomitant injuries that could affect upper-limb function; moreover, 4 patients were excluded for delayed surgery and 2 because they sustained polytrauma.

### 2.1. Surgical Technique

All procedures were performed under general anesthesia with the patient positioned in a semi-seated (beach-chair) configuration. In both centers, antegrade intramedullary nailing was adopted as the standard fixation method, but the surgical approach differed slightly according to institutional protocols and implant type.

At the Guglielmo da Saliceto Hospital (Piacenza), fixation was performed using the Diphos^®^ long nail (LimaCorporate, San Daniele del Friuli, Italy). The approach was typically percutaneous or mini-open, with the entry point placed more medially, close to the muscular portion of the supraspinatus, to minimize cuff damage. Temporary fixation with one or more Kirschner wires was used to manipulate and rotate the humeral head as a single fragment, optimizing three-dimensional alignment before nail insertion. Additional screws were inserted to stabilize tuberosity fragments and to support the calcar region when necessary.

At the Circolo Hospital (Varese), fixation was performed using the Trigen^®^ Humeral Nail (Smith & Nephew, Memphis, TN, USA). Through an incision starting from the anterolateral margin of the acromion, the deltoid fibers were split along their orientation to expose the raphe separating the anterior and middle heads, while carefully protecting the anterior branch of the axillary nerve running a few centimeters distal to the greater tuberosity. After subacromial bursectomy, the supraspinatus tendon was incised to expose the humeral head, and the entry point was prepared under fluoroscopic control. Fracture reduction and nail insertion were then performed using the beach-chair position with intraoperative imaging guidance.

In both centers, distal locking was performed under fluoroscopic control using one or two cortical screws according to bone stock and fracture configuration. Locking mode (static or dynamic) was selected at the discretion of the surgeon based on fracture patterns and stability requirements. This variability reflected standard institutional practice in both centers. Final implant positioning and screw orientation were verified in orthogonal projections before wound closure, and the rotator cuff was repaired when incised.

### 2.2. Postoperative Course

After surgery, patients remained hospitalized for a few days before being discharged home. The operated limb was immobilized in a deep pocket arm sling for three to four weeks, depending on the protocol of the treating center, and could only be removed for dressing, hygiene, and early assisted elbow mobilization. Physiotherapy was then initiated, starting with passive and active mobilization in all planes and followed by muscle strengthening exercises, in accordance with radiographic evidence of fracture consolidation. A gradual return to work was subsequently planned.

Stitches were removed between 12 and 15 days postoperatively. Outpatient follow-up was carried out monthly for the first three months and subsequently at six and twelve months. Each follow-up visit included clinical and radiographic evaluation with anteroposterior and lateral X-rays, which were compared with preoperative images to assess healing and detect complications. Clinical examination focused on range of motion and muscle strength, recorded on standardized forms.

At one-year follow-up, functional outcomes were assessed with the Disabilities of the Arm, Shoulder and Hand (DASH) score, the Oxford Shoulder Score (OSS), the Constant Shoulder Score (CSS) for both upper limbs, and the Visual Analogue Scale (VAS). During the entire follow-up, special attention was paid to the occurrence of complications such as neurological deficits, wound healing disorders, infections, nonunion, and implant-related issues.

### 2.3. Statistical Analysis

Data were analyzed using JASP (Version 0.17.1; JASP Team, 2023). Descriptive statistics were applied to summarize patient characteristics and outcomes. Differences in functional scores according to fracture type, time to surgery, and comorbidities were assessed using analysis of variance (ANOVA). Comparisons between sex, diabetes status, and osteoporosis were performed with Student’s t-test. Correlations between age and functional scores were evaluated with Spearman’s rho. A multivariate analysis was conducted to determine the influence of demographic characteristics on functional outcomes. Finally, linear regression analysis was used to identify significant correlations between variables and cofactors.

Multivariate models included age, sex, fracture level, diabetes, osteoporosis, and time-to-surgery as covariates. Regression coefficients (β) with 95% confidence intervals were calculated for each variable, and model fit was evaluated using the adjusted R^2^ statistic. Time-to-surgery emerged as the only independent predictor significantly associated with DASH, Oxford, and Constant scores (*p* < 0.01), confirming the results of the ANOVA and post-hoc analyses. No significant multicollinearity was detected among variables (VIF < 2.0). Center-level comparisons used t-tests or Mann–Whitney U for continuous variables and χ^2^ or Fisher’s exact for categorical variables.

The level of statistical significance was set at *p* < 0.05.

## 3. Results

A total of 238 patients were initially considered eligible for the study. After applying the exclusion criteria, 202 patients were included in the final cohort. The mean follow-up was 57.7 ± 19.6 months (range 24.3–92.5).

In Table 1 all the main information about the cohort is presented.

When stratifying the mechanism of injury by age, high-energy trauma (motor vehicle accidents, sports, or falls from height) was most frequent in patients younger than 40 years and progressively decreased across the 40–60 and 60–80-year groups, becoming rare in those older than 80 years. Conversely, low-energy mechanisms (simple/domestic falls) predominated in the 60–80 and >80-year groups (Table 2).

Clinical records showed comparable comorbidity profiles between the two centers (Table 1). Diabetes was present in 12.9% and osteoporosis in 15.3% of patients, while obesity and smoking were less frequent. Early postoperative complications within 30 days occurred in 5.9% of cases, primarily minor wound irritation or transient shoulder pain.

The relationship between sex, age, and mechanism of injury is summarized in Table 2. High-energy trauma predominated among younger males, whereas low-energy domestic falls were the leading cause of fractures in older women. Specifically, over 80% of patients younger than 40 years sustained high-energy injuries, compared with fewer than 10% of those older than 70 years, confirming the typical bimodal epidemiologic distribution of humeral shaft fractures.

In total, 142 patients (70.3%) received a Diphos^®^ long nail (LimaCorporate, San Daniele del Friuli, Italy), while 60 patients (29.7%) were treated with a Trigen^®^ humeral long nail (Smith & Nephew, Memphis, TN, USA). No significant differences were detected between centers for age, sex distribution, trauma mechanism, fracture level distribution, or time-to-surgery (all *p* > 0.05).

At one-year follow-up, functional assessment showed a mean Oxford Shoulder Score of 39.0 ± 8.9 (range 12–48), a mean DASH score of 16.6 ± 16.1 (range 0.0–68.2), a mean Constant Score of 73.5 ± 20.1 (range 25–100), and a mean VAS score of 0.9 ± 1.7 (range 0–6). When comparing the Constant Score between the operated and contralateral limbs, outcomes were classified as poor in 9 cases (4.5%), fair in 12 (5.9%), good in 35 (17.3%), and excellent in 146 (72.3%). Table 3 shows the average scores of the cohort.

When patients were stratified by age (<40, 40–60, 60–80, and >80 years), no statistically significant differences were observed in DASH, OSS, Constant, or VAS scores (all *p* > 0.05) (Table 4). These results confirm that functional recovery after antegrade intramedullary nailing was comparable across age groups.

At the end of follow-up, 22 complications were observed: 8 cases of nerve injury (4.0%), 4 cases of rotator cuff injury (2.0%), 3 cases of pseudoarthrosis (1.5%), 2 wound infections (1.0%), 2 cases of proximal screw protrusion (1.0%), and 1 case of distal screw mobilization (0.5%) (Table 5).

**Figure 1:**
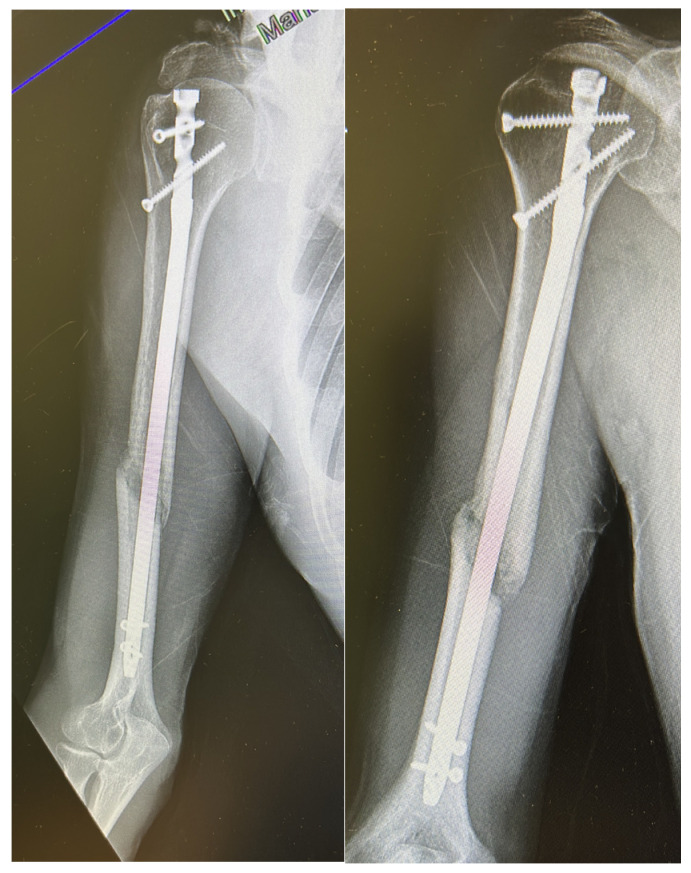
Radiographic example of humeral shaft nonunion after antegrade intramedullary nailing. The images obtained 9 months postoperatively show a persistent fracture line with no cortical bridging or callus formation, consistent with atrophic nonunion.

When complications were analyzed according to fracture location, radial nerve injuries were more frequent in distal-third fractures (5/42; 11.9%) compared with middle-third fractures (3/120; 2.5%) (*p* = 0.049), while no cases occurred in proximal-third fractures. Other complications, such as screw mobilization or delayed union, showed no clear association with fracture level. A representative case of atrophic nonunion observed nine months after antegrade intramedullary nailing is presented in Figure 1, illustrating the radiographic features of persistent fracture line and absent callus formation.

Among the 202 patients, 8 (4.0%) presented radial nerve symptoms prior to surgery. All cases were identified on admission and classified as primary nerve injuries, with no new or secondary palsies occurring postoperatively. Of these, 6 patients (3.0%) had isolated sensory loss in the radial nerve distribution, and 2 patients (1.0%) exhibited motor involvement (wrist and finger extension weakness). All lesions were consistent with neurapraxia or axonotmesis. At 6-month follow-up, six patients (75%) achieved full neurological recovery, while two (25%) had persistent mild sensory deficit without motor impairment.

To assess whether implant design influenced complication rates, a subgroup analysis was performed comparing patients treated with the Diphos^®^ nail (n = 142) and the Trigen^®^ humeral nail (n = 60). The overall complication rate was 8.4% in the Diphos^®^ group and 6.6% in the Trigen^®^ group (*p* = 0.74, Fisher’s exact test), showing no statistically significant difference. The distribution of complication types (nerve injury, pseudoarthrosis, screw protrusion, or wound infection) was similar between implants. Functional outcomes did not differ significantly between the two groups in terms of DASH, OSS, Constant, or VAS scores (all *p* > 0.05).

Statistical analysis demonstrated a significant association between time to surgery (TTS) and functional outcomes. ANOVA revealed significant differences in DASH, Oxford and Constant scores at one year among patients operated within 24 h, within 48 h, or after 48 h. Post hoc Games-Howell tests confirmed that patients treated within 24 or 48 h had significantly better results compared to those treated after 48 h. Mean DASH values were 15.0, 10.0, and 24.2 respectively (*p* < 0.01). Mean Oxford scores were 42.5 and 40.5 for the <24 h and 24–48 h groups, compared to 35.4 for the >48 h group (*p* < 0.01). Mean Constant scores were 78.6 and 81.7 in the early groups versus 66.7 in the >48 h group (*p* < 0.01) (Table 6). Beyond time-to-surgery, functional outcomes did not differ by sex, diabetes, or osteoporosis (all *p* > 0.05). Fracture level (proximal/middle/distal third) showed no significant association with DASH, OSS, or Constant scores on ANOVA. Age displayed weak correlations with outcomes (higher age associated with slightly worse OSS/Constant and higher DASH), consistent with the multivariate results.

Conversely, ANOVA found no significant differences in outcomes according to fracture pattern or comorbidities. Student’s t-test revealed significantly worse results in patients who experienced complications, with mean DASH, Oxford and Constant scores of 23.3, 35.5 and 67.5, compared to 15.1, 40.2 and 77.4 in those without complications (*p* < 0.05). No significant differences were found when comparing scores between sexes, nor between diabetic and osteoporotic patients.

Spearman’s rho correlation analysis showed a weak but significant negative correlation between age and both Oxford (rho = −0.18, *p* < 0.01) and Constant scores (rho = −0.23, *p* < 0.01), and a weak positive correlation between age and DASH score (rho = 0.124, *p* = 0.03).

To assess the functional impact of postoperative complications, the Constant scores of affected and unaffected shoulders were compared. The mean difference between the injured and contralateral limb was 24.8 points in patients with complications, compared to only 6.0 points in those without (*p* = 0.011). This difference indicates a clinically meaningful reduction in shoulder function among patients who developed adverse events.

## 4. Discussion

Our study confirms the safety and efficacy of antegrade intramedullary nailing (IMN) for the treatment of humeral shaft fractures (HSFs), with good functional recovery and a low incidence of complications. The absence of significant differences in functional outcomes across fracture patterns, as assessed by ANOVA tests, suggests that this technique can be safely applied to both proximal and middle-third diaphyseal fractures, as well as combined fractures involving the neck. Procedure length did not affect outcomes, indicating that surgical duration is not a critical variable in predicting functional recovery.

The demographic distribution of our cohort is in line with previous literature. The mean age of female patients was significantly higher (64.8 years) than that of males (46.5 years), with a predominance of women in the elderly group. These findings mirror the age clusters described by Pidhorz et al., who reported two peaks: 20–30 years for men, usually associated with high-energy trauma such as motor vehicle accidents, and 60–70 years for women, often affected by osteoporosis and injured after low-energy falls [19]. Interestingly, in our cohort, the mean age of men was lower than that reported in other series, which may reflect differences in trauma mechanisms.

Clinical and functional outcomes were encouraging. Almost two-thirds of our patients achieved good or excellent results in all functional scores, confirming the reliability of this technique. Our Constant scores showed excellent or good results in more than 70% of patients, a finding comparable to that reported by Rommens et al., who observed excellent outcomes in 91.3% of cases [21]. In addition to statistical significance, the observed improvements in functional scores exceeded established minimal clinically important difference (MCID) thresholds reported in the literature, supporting the clinical relevance of the findings. Specifically, the mean DASH score in our cohort (16.6 ± 16.1) was well below the MCID threshold of approximately 10 points for improvement after upper-limb interventions, while the mean Oxford Shoulder Score (39.0 ± 8.9) surpassed the MCID range of 4–6 points typically considered meaningful in postoperative recovery. These results indicate that the functional benefits observed after antegrade intramedullary nailing are not only statistically but also clinically significant, reinforcing the therapeutic value of this approach. The mean Oxford Shoulder Score and DASH values were consistent with those described in previous series, confirming the reproducibility of our results.

At follow-up, the mean VAS pain score was 0.93, with 11.7% of patients reporting residual pain ≥ 3. This result is slightly better than that described by Baltov et al., who reported persistent shoulder pain in 16.2% of patients treated with first- and second-generation antegrade nails [22]. Christ et al. investigated a rotator cuff–sparing approach through the rotator interval combined with biceps tenodesis and reported a mean VAS of 0.8 at 3 months, concluding that the intra-articular portion of the long head of the biceps may often contribute to residual pain after surgery [23]. In our study, VAS was significantly higher in patients with diabetes and osteoporosis, with mean scores of 1.77 and 1.82, respectively, compared to 1.41 and 1.09 in patients without these comorbidities, suggesting that systemic conditions negatively influence pain recovery.

The surgical approach represents a key factor in functional outcomes. In our cohort, all patients were treated with the classical anterolateral access, which requires splitting the supraspinatus tendon to insert the nail at the medial sulcus of the greater tuberosity. While this approach is widely adopted, it carries the risk of rotator cuff damage and subacromial impingement due to implant prominence. Several alternative approaches have been proposed. Park et al. described a rotator interval entry point that improved functional results but carried the risk of axillary nerve injury during proximal screw placement [24]. Dimakopoulos et al. proposed a lateral entry through the greater tuberosity, which spared the cuff but increased the risk of cortical fracture and greater tuberosity fragmentation [25]. Despite these alternatives, the outcomes in our series suggest that with meticulous surgical technique and early rehabilitation, the traditional approach remains safe and effective.

Regarding fracture distribution, our results were comparable with those reported in the literature. Proximal third fractures accounted for 33.3%, middle third for 28.3%, combined for 35%, and distal third for 3.3%, figures consistent with the review by Pidhorz [19]. A higher proportion of radial nerve injuries was observed in distal-third fractures compared with middle-third fractures (11.9% vs. 2.5%; *p* = 0.049), consistent with the known anatomical vulnerability of the nerve in the distal humeral region. This pattern likely reflects primary lesion mechanisms rather than iatrogenic injury and supports the importance of careful preoperative assessment in distal fractures. No significant differences in functional recovery or surgical complication rates were observed according to fracture location, although the very limited number of distal-third fractures in our cohort warrants caution.

The overall complication rate in our series was 9.9%, with radial nerve palsy (4.0%) and rotator cuff injury (2.0%) being the most frequent. Radial nerve palsy was present preoperatively in all cases and manifested predominantly as sensory loss in the nerve’s distribution. Our incidence is comparable to, or even lower than, that reported in the literature, where rates of radial nerve involvement of around 10–12% have been described [26]. Pseudoarthrosis occurred in 1.6% of cases, a rate equivalent to the 1.3% reported by Gottschalk et al., and no infections were observed, whereas other series reported rates between 0 and 6% [27]. These data reinforce the safety of IMN, especially with modern nails and standardized postoperative care.

When comparing the two implants used across centers, no significant differences emerged in either complication profile or functional outcomes. Both Diphos^®^ and Trigen^®^ nails demonstrated comparable safety and efficacy, suggesting that implant design had minimal influence on nerve injury rate, screw migration, or overall shoulder function. These findings support the notion that surgical technique and timing, rather than specific implant type, are the main determinants of outcome after antegrade humeral nailing.

A notable finding in our series is the association between time to surgery and functional recovery. Patients operated within 48 h achieved significantly greater shoulder flexion (165.6° vs. 141°) compared to those treated later, and this difference was reflected in functional scores such as Oxford and Constant. Although the mean DASH scores differed significantly across surgical timing groups, the distribution was not strictly linear, with the 24-48 h group showing slightly lower values than the <24 h cohort. This likely reflects a more balanced perioperative course in patients operated within this interval, when early soft-tissue recovery and optimal surgical timing coincide. From a clinical standpoint, a DASH score above 10 generally corresponds to at least mild functional disability in upper-limb activities; therefore, the average values observed in our series indicate good recovery and only minimal residual limitations in most patients. This result highlights the importance of early (but not necessarily emergent) intervention in optimizing outcomes, although further prospective studies are needed to confirm this observation.

Unlike Verdano et al., who reported worse Constant scores in patients older than 60 years [28], we did not observe age-related differences in outcomes. However, the presence of complications significantly affected recovery: the mean difference in Constant score between the injured and contralateral limb was 24.8 in patients with complications, compared to only 6.0 in those without (*p* = 0.011). This emphasizes the impact of avoiding perioperative complications to ensure optimal results.

Finally, our study confirms the validity of using Oxford, DASH, and Constant scores in assessing shoulder function after IMN, as these instruments provided consistent and reproducible evaluations within and across patient groups.

Recent evidence comparing antegrade intramedullary nailing and plate fixation for humeral shaft fractures remains heterogeneous but broadly indicates that both techniques achieve reliable union, with trade-offs in complications and function. Contemporary meta-analyses and randomized-data syntheses report similar union rates between methods, while plating may shorten time to union and improve short- to mid-term shoulder function at the cost of higher rates of iatrogenic radial nerve palsy and reoperation; conversely, nailing tends to preserve biology with lower soft-tissue disruption but can be associated with postoperative shoulder pain and range-of-motion limitations related to entry and proximal implant prominence [29,30,31].

Network and conventional meta-analyses published in 2024–2025 further refine these signals: pooled estimates suggest comparable overall complication profiles between intramedullary nailing and plate fixation, with a tendency toward fewer nerve injuries after nailing but marginally better early functional scores after plating; importantly, no consistent difference has emerged in nonunion risk. These data provide an external benchmark for the present cohort, in which antegrade nailing yielded high rates of good-to-excellent functional recovery and a low complication burden at long-term follow-up. Within this context, our finding that surgery within 48 h independently correlates with superior DASH, OSS, and Constant outcomes supports the interpretation that timely fixation is a modifiable determinant of recovery, irrespective of implant choice, and adds clinically actionable nuance to the current comparative literature [32,33].

In line with these observations, the outcomes reported in the present series mirror the results of the most recent comparative studies and meta-analyses, which have shown no major differences in union or overall complication rates between nailing and plating [30,32]. Plating may allow faster early recovery but carries a higher risk of iatrogenic radial nerve palsy, whereas antegrade nailing provides equivalent long-term function with less soft-tissue disruption [32]. These findings collectively support antegrade IMN as a safe, effective, and reproducible option for humeral shaft fracture fixation.

### Strengths and Limitations

The main strengths of this study are the relatively large sample size and the long follow-up, which averaged nearly five years with a minimum of two years for all patients. The bicenter design improves the external validity of the findings, while the use of standardized functional outcome measures ensures comparability with previous studies.

This study also presents several limitations. Its retrospective design may have introduced selection and information bias, and surgeries were performed by multiple operators with variable levels of experience, which could have influenced intraoperative events such as screw prominence or transient nerve irritation. Although both centers followed broadly similar surgical and rehabilitation principles, minor differences in perioperative management and physiotherapy timing cannot be excluded and may have contributed to variability in functional recovery. Furthermore, the absence of a comparison group treated with plate fixation prevented a direct evaluation of the relative efficacy of intramedullary nailing versus alternative fixation methods. Some follow-up assessments were conducted during the COVID-19 pandemic through remote or telephone-based questionnaires, potentially affecting the precision of functional evaluations [34,35]. Finally, the lack of pre-injury baseline scores limited the ability to quantify recovery relative to each patient’s previous functional status.

Despite these limitations, the findings indicate that antegrade intramedullary nailing is a safe and effective treatment for humeral shaft fractures. The low complication rate and the high proportion of excellent or good functional outcomes support its widespread adoption. The significant association between shorter time to surgery and improved results underscores the importance of early surgical intervention whenever feasible. Nonetheless, treatment should remain individualized, taking into account patient age, comorbidities, and functional demands.

## 5. Conclusions

Intramedullary nailing represents a safe and effective option for the treatment of humeral shaft fractures, with high functional recovery rates and low complication incidence. Early surgical intervention appears to play a key role in improving outcomes, particularly in terms of pain and range of motion. Further prospective studies with larger cohorts are warranted to confirm these findings and to optimize surgical strategies.

## Figures and Tables

**Table 1:** Demographic and baseline characteristics of the cohort. No statistically significant differences were observed between the two centers in terms of demographic characteristics, fracture distribution, or time-to-surgery (all *p* > 0.05). This confirms the comparability of the two institutional cohorts.

	Total (n = 202)	Circolo Hospital (n = 60)	Guglielmo da Saliceto Hospital (n = 142)
Men	89 (44.1%)	38 (63.3%)	51 (35.9%)
Women	113 (55.9%)	22 (36.6%)	91 (64.1%)
Mean age (years)	61.0 ± 15.2	58.1 ± 17.4	62.2 ± 14.0
Min–Max (years)	21–79	16–79	18–79
Side, right	116 (57.5%)	27 (45.0%)	59 (41.5%)
Side, left	86 (42.5%)	33 (55.0%)	83 (58.5%)
Dominant side	112 (55.4%)	43 (71.7%)	69 (48.9%)
High energy trauma	56 (27.7%)	17 (28.3%)	39 (27.5%)
Low energy trauma	146 (82.3%)	43 (71.7%)	103 (72.5%)
Proximal third fractures	97 (48.0%)	24 (40.0%)	73 (51.4%)
Middle third fractures	78 (38.6%)	22 (36.7%)	56 (39.4%)
Distal third fractures	9 (4.5%)	2 (3.3%)	7 (4.9%)
Multifragmentary fractures	18 (8.9%)	12 (20.0%)	6 (4.2%)
Mean TTS (days)	2.5 ± 1.9	2.4 ± 1.9	2.5 ± 1.9
Range TTS (days)	0–15	0–10	0–15
Procedure length	63.4 min	72.3 min	59.6 min
Diabetes mellitus	26 (12.9%)	8 (13.3%)	18 (12.7%)
Osteoporosis	31 (15.3%)	9 (15.0%)	22 (15.5%)
Obesity (BMI > 30)	19 (9.4%)	5 (8.3%)	14 (9.9%)
Smokers	48 (23.8%)	14 (23.3%)	34 (23.9%)
Early complications	12 (5.9%)	3 (5.0%)	9 (6.3%)

**Table 2:** Distribution of trauma mechanism by age group and sex.

Age	Male	Female	High-Energy Trauma	Low-Energy Trauma	Main Causes of Injury
<40 y	26 (12.9%)	8 (4.0%)	30 (83.3%)	4 (11.1%)	Sports injury
40–59 y	31 (15.3%)	19 (9.4%)	32 (64.0%)	18 (36.0%)	Road traffic accident
60–79 y	21 (10.4%)	47 (23.3%)	10 (15.6%)	58 (90.6%)	Domestic falls
≥80 y	11 (5.4%)	10 (5.0%)	1 (5.0%)	20 (95.0%)	Low-energy fall

**Table 3:** Functional outcomes at one-year follow-up. Reference ranges and interpretation: Oxford Shoulder Score (0–48, with higher values indicating better shoulder function; 0–19 = poor, 20–29 = fair, 30–39 = good, ≥40 = excellent). Disabilities of the Arm, Shoulder and Hand (DASH) score (0–100, with lower values indicating better upper-limb function; 0–10 = excellent, 11–20 = good, 21–40 = fair, >40 = poor). Constant Shoulder Score (0–100, with higher values indicating better outcome; <70 = poor, 70–79 = fair, 80–89 = good, ≥90 = excellent). Visual Analogue Scale (VAS) for pain (0–10, with lower values indicating less pain; 0–1 = no or minimal pain, 2–4 = mild, 5–7 = moderate, ≥8 = severe).

Score	Mean ± SD	Min	Max
Oxford	39.0 ± 8.9	12	48
DASH	16.6 ± 16.1	0	68.2
VAS	0.9 ± 1.7	0	6
Constant	73.5 ± 20.1	25	100

**Table 4:** Functional outcomes according to age group. Values are expressed as mean ± standard deviation. *p*-values obtained using one-way ANOVA across age groups.

Age	n.	DASH (±SD)	OSS (±SD)	Constant (±SD)	VAS (±SD)
<40 y	34	14.3 ± 11.8	45.2 ± 7.6	85.5 ± 9.3	1.0 ± 1.6
40–59 y	50	15.8 ± 13.9	44.0 ± 8.1	84.2 ± 9.8	0.9 ± 1.7
60–79 y	88	17.1 ± 15.7	43.1 ± 8.6	82.8 ± 10.4	1.1 ± 1.8
>80 y	30	18.4 ± 17.2	42.5 ± 9.2	81.6 ± 11.2	1.2 ± 1.9
*p*-value		0.39	0.27	0.31	0.58

**Table 5:** Complications at final follow-up.

Complication	Cases	Incidence
Radial nerve injury	8	4.0%
Rotator cuff injury	4	2.0%
Pseudoarthrosis	3	1.5%
Wound infection	2	1.0%
Proximal screw protrusion	2	1.0%
Distal screw mobilization	1	0.5%

**Table 6:** Functional outcomes by time to surgery (TTS). *p*-values from one-way ANOVA; pairwise differences tested with Games–Howell post-hoc.

TTS	DASH	Oxford	Constant
<24 h	15.0	40.5	78.6
24–48 h	10.0	42.5	81.7
>48 h	24.2	35.4	66.7
*p*-value	<0.01	<0.01	<0.01

## Data Availability

The original data presented in this study are available under request to the corresponding author.
